# Association between endogenous oxytocin levels and live birth rates following fresh embryo transfer: a prospective cohort study

**DOI:** 10.3389/fendo.2026.1850346

**Published:** 2026-06-02

**Authors:** Ruiqiong Zhou, Zhaoyi Wang, Zhenghong Zhu, Mei Dong, Jiao Wang, Li Huang, Quan Qi, Xiqian Zhang, Fenghua Liu

**Affiliations:** 1 Center for Reproductive Medicine, Guangdong Women and Children Hospital, Guangzhou, Guangdong, China; 2 Women and Children’s Hospital, Southern University of Science and Technology, Shenzhen, China; 3 School of Public Health, Sun Yat-sen University, Guangzhou, China

**Keywords:** fresh embryo transfer, *in vitro* fertilization, live birth rate, ovarian response, oxytocin

## Abstract

**Objective:**

To investigate the associations between serum and follicular fluid oxytocin levels and live birth rate (LBR) in IVF cycles, and to examine their correlations with clinical parameters.

**Design:**

Prospective cohort study.

**Methods:**

A total of 680 infertile patients undergoing IVF cycles between October 2024 and January 2025 were enrolled for serum and follicular fluid oxytocin measurement. The associations between serum and follicular fluid oxytocin levels and LBR were assessed using three statistical approaches: multivariable logistic regression, inverse probability weighting, and a double robust model. Smoothed curve fitting was employed to explore the relationships between oxytocin levels and LBR. Additionally, Spearman correlation analysis was performed to examine the associations between oxytocin levels and key clinical parameters.

**Results:**

Serum oxytocin demonstrated a negative linear association with LBR (p-nonlinear = 0.911), whereas follicular fluid oxytocin showed a positive association (p-nonlinear = 0.425). In the double robust model, each 100 pg/mL increase in serum oxytocin decreased the likelihood of live birth by 37% (95% CI: 25%–47%), whereas a corresponding increase in follicular fluid oxytocin improved it by 18% (95% CI: 2%–37%). Additionally, serum oxytocin correlated positively with triglycerides (r = 0.294), LDL-C (r = 0.165), and insulin (r = 0.257), but negatively with HDL-C (r = –0.167). Conversely, follicular fluid oxytocin correlated positively with ovarian response markers, including hCG-trigger day estradiol (r = 0.229) and oocytes retrieved (r = 0.248).

**Conclusion:**

Higher basal serum oxytocin prior to ovarian stimulation is negatively associated with LBR following fresh embryo transfer, whereas elevated follicular fluid oxytocin correlates with improved ovarian response.

## Introduction

Despite substantial advances in assisted reproductive technology (ART) over the past two decades, live birth rates (LBRs) per *in vitro* fertilization (IVF) cycle remain around 30% (1). Embryo transfer is a crucial step, with its success primarily influenced by embryo quality, endometrial receptivity, and maternal environment (2).

Oxytocin, synthesized in the hypothalamus and secreted by the posterior pituitary, plays key roles in parturition, lactation, social behavior, and metabolism (3). Furthermore, it is produced locally in reproductive tissues such as the ovary and uterus. Granulosa-luteal cells express oxytocin mRNA and protein, with concentrations increasing during the luteal phase, suggesting a regulatory role in ovarian function (4, 5). Likewise, the uterus produces oxytocin in a paracrine or autocrine manner, particularly during peri-implantation and parturition (6, 7). Oxytocin acts through the oxytocin receptor (OXTR), a G protein–coupled receptor widely distributed across tissues including the ovary, uterus, testis, vasculature, heart, pancreas, and kidney (7).

Oxytocin is well recognized for regulating uterine contractions. While moderate uterine activity may facilitate embryo implantation, excessive contractions can negatively affect IVF outcomes (8, 9). Although oxytocin antagonists can inhibit uterine contractions by competitively binding to OXTR, studies evaluating their effects on implantation rates have produced inconsistent results (10).

Despite the critical role of oxytocin in reproductive physiology, the clinical significance of baseline endogenous oxytocin levels in IVF outcomes remains largely unexplored. Currently, clinical attention has predominantly focused on pharmacological interventions, such as oxytocin antagonists, to suppress uterine contractions during embryo transfer. However, the inconsistent results from these clinical trials highlight a knowledge gap regarding patients’ systemic oxytocin profiles. To date, understanding of systemic oxytocin is limited to a single small-scale study (n=108) investigating serum levels in patients with repeated implantation failure, which observed a non-significant trend of elevation without definitive conclusions (11). Moreover, while oxytocin is known to be synthesized locally in the ovary, its specific concentration within the follicular fluid microenvironment and its potential correlation with ovarian response and embryo viability have yet to be fully elucidated.

Beyond its well-established mechanical effects on the myometrium, oxytocin is increasingly recognized for its role in systemic metabolic regulation. Because metabolic homeostasis is critical for optimal endometrial receptivity and the window of implantation, characterizing baseline oxytocin profiles may yield critical insights into the biochemical environment necessary for reproductive success. Accordingly, this study aimed to evaluate baseline serum and follicular fluid oxytocin levels to determine their associations with live birth rates following fresh embryo transfer. We hypothesized that systemic and local oxytocin concentrations exert distinct, compartment-specific effects on IVF outcomes. Specifically, we sought to investigate whether baseline circulating oxytocin serves as a proxy for the maternal systemic environment, while follicular fluid oxytocin indicates the quality of the follicular microenvironment and subsequent oocyte competence. By evaluating these two physiological compartments, we ultimately sought to determine whether endogenous oxytocin profiles offer predictive value for reproductive success.

## Materials and methods

### Study design and participants

This prospective observational cohort study was conducted at a tertiary reproductive medicine center between October 2024 and January 2025. The study was approved by the Institutional Review Board of Guangdong Women and Children Hospital, and written informed consent was obtained from all participants. Women undergoing IVF or intracytoplasmic sperm injection (ICSI) were prospectively enrolled for measurement of oxytocin levels in serum and follicular fluid during their treatment cycle. Exclusion criteria included: (1) recent hormonal therapy; (2) uterine abnormalities (e.g., intrauterine adhesions, septate uterus, or submucosal fibroids); (3) psychiatric disorders or current use of antidepressant or anxiolytics; (4) comorbidities (e.g., autoimmune diseases, renal insufficiency, hypertension, diabetes mellitus, or malignancy); (5) preimplantation genetic testing (PGT); (6) recurrent pregnancy loss or RIF; (7) missing core clinical parameters. Patients were stratified into quartiles based on serum oxytocin levels measured during the menstrual phase prior to ovarian stimulation: < 25th percentile (13.9-129.8 pg/mL), 25th-50th percentile (130.1-175.2 pg/mL), 51th-75th percentile (175.4-240.4 pg/mL), and > 75th percentile (241.6-868.7 pg/mL).

### Sample collection and measurement

Fasting blood samples were collected in the morning (between 08:00 and 10:00) on day 2 or 3 of the menstrual cycle prior to ovarian stimulation. Follicular fluid was obtained on the day of oocyte retrieval, approximately 35–36 hours following the administration of the final oocyte maturation trigger. Only clear fluid aspirates from dominant follicles (18–20 mm in diameter) without visible blood or flushing buffer contamination were collected. All samples were stored at −80 °C for an average of two months until analysis. Oxytocin concentrations were measured using a commercial ELISA kit (Enzo Life Sciences, Farmingdale, NY, USA) after extraction with HyperSep C18 solid-phase extraction columns (Thermo Fisher Scientific, Waltham, MA, USA) according to the manufacturer’s protocol. Optical density was read on a microplate reader (Thermo Fisher Scientific).

Serum FSH, LH, estradiol, and progesterone were quantified by electrochemiluminescence immunoassay on the Roche Elecsys 2010 analyzer (Roche Diagnostics, Mannheim, Germany). AMH and fasting insulin were measured by chemiluminescence immunoassay. Fasting glucose, triglycerides, and total cholesterol were determined enzymatically, while low-density lipoprotein cholesterol (LDL-C) and high-density lipoprotein cholesterol (HDL-C) were assessed by colorimetric methods.

### Ovarian stimulation

Ovarian stimulation was performed using a flexible GnRH antagonist protocol. Recombinant FSH (Gonal-f, Merck Serono, or Puregon, MSD/Organon) was initiated at 100–300 IU/day on cycle day 2 or 3, with dose adjustments based on ovarian response monitored by transvaginal ultrasound and serum hormone levels every 3–4 days. GnRH antagonist (ganirelix, 0.25 mg/day; MSD/Organon) was started when the leading follicle reached ≥12 mm and continued until hCG trigger. Final oocyte maturation was triggered with 6,000–10,000 IU hCG when ≥3 follicles reached ≥17 mm or ≥2 follicles reached ≥18 mm. Oocyte retrieval was performed 35–36 hours later via transvaginal ultrasound-guided follicle aspiration.

### Embryo culture and transfer

Fertilization was performed according to semen parameters, with normal fertilization (2PN) confirmed 16–18 hours post-insemination. Two independent embryologists graded Day 3 cleavage-stage embryos (12) and Day 5–6 blastocysts using the Gardner system (13). Depending on embryo quality and patient preference, either two Day 3 cleavage embryos or a single Day 5 blastocyst (≥3BC) were transferred. A freeze-all strategy was applied for severe ovarian hyperstimulation syndrome (OHSS), abnormal endometrial morphology, serum progesterone ≥1.5 ng/mL or no transferable embryos. Luteal phase support started one day post-retrieval with either intramuscular progesterone (40 mg/day) or vaginal gel (90 mg/day) plus oral dydrogesterone (10 mg twice daily), continuing until 10 weeks of gestation.

### Outcome measures

The primary outcome was live birth rate, defined as the delivery of a live-born infant after 24 weeks’ gestation. Secondary outcomes included implantation rate (gestational sacs per transferred embryo), biochemical pregnancy (serum β-hCG >10 IU/L at 14 days post-transfer), clinical pregnancy (intrauterine gestational sac at 5 weeks), and pregnancy loss (loss before 24 weeks). Obstetrical and neonatal outcomes comprised singleton birth, newborn sex, gestational age, delivery mode, preterm birth (<37 weeks), and birthweight metrics [including Z-scores, low birthweight (<2500g), macrosomia (>4000g), and <10th/>90th percentiles]. Birthweight Z-scores were adjusted for gestational age and sex using Chinese singleton references (14, 15). Cycle outcomes evaluated oocytes retrieved, numbers and rates of 2PN zygotes, usable cleavage embryos, and blastocysts, alongside moderate-to-severe OHSS incidence (16).

### Statistical analysis

Analyses were performed using SPSS (version 22.0; IBM Corp.) and R (version 4.3). A two-sided P < 0.05 was considered statistically significant. Data distribution was assessed using the Kolmogorov-Smirnov test. Continuous variables were expressed as mean ± SD or median (interquartile range) and compared using Student’s t-test, one-way ANOVA, the Mann-Whitney U test, or the Kruskal-Wallis test, as appropriate. Categorical variables were presented as frequencies (percentages) and compared using χ² test or Fisher’s exact test.

#### Rationale for advanced modeling strategy

Conventional multivariable regression modeling primarily addresses measured confounding but is limited in handling potential selection bias. In this cohort, a substantial proportion of enrolled patients (52.9%) did not proceed to fresh embryo transfer due to predefined freeze-all strategy (e.g., high OHSS risk or elevated progesterone). Analyzing only the transferred subpopulation would introduce significant selection bias, compromising the generalizability of the live birth outcomes to the broader IVF population.

To robustly evaluate the independent associations between oxytocin levels (serum and follicular fluid) and LBR, we implemented a sequential, three-tier modeling strategy to systematically address both confounding and selection biases:

- Model 1 (Multivariable Logistic Regression): A standard binary logistic regression model was constructed to adjust for baseline confounding. Covariates were selected based on baseline differences (*P* < 0.10) and clinical relevance (**Supplementary Table 1**), incorporating age, BMI, AMH, causes of infertility, fasting insulin, duration of stimulation, endometrial thickness, serum estradiol and progesterone levels on hCG trigger day, and number of embryos transferred.
- Model 2 (Inverse Probability Weighting, IPW): Conceptually, IPW functions by assigning specific statistical weights to each patient to create a balanced pseudo-population where the likelihood of undergoing fresh transfer is independent of baseline variables, effectively simulating a randomized environment. To mitigate the selection bias introduced by transfer cancellations, Model 2 utilized IPW conditional on transfer-predictive covariates to balance the baseline distributions between patients who did and did not undergo fresh transfer.
- Model 3 (Double Robust Model, DRM): To maximize the validity of our causal inferences, a DRM was applied to simultaneously adjust for selection bias and exposure-related confounding. The primary methodological advantage of DRM is its built-in protection against model misspecification. The final composite weight was derived by combining the selection weights (from Model 2) and the inverse probability of exposure weights. This approach yields unbiased and highly reliable effect estimates provided that either the selection model or the outcome model is correctly specified.

Detailed mathematical formulations and weight derivations for the IPW and DRM approaches are provided in the **Supplementary Methods**.

Additionally, restricted cubic spline smoothed curve fitting was employed to explore potential non-linear relationships between oxytocin levels and LBR. Spearman correlation analysis was utilized to assess the relationships between oxytocin concentrations and continuous clinical parameters.

Given the prospective, exploratory nature of characterizing endogenous oxytocin profiles in relation to fresh transfer outcomes, a formal *a priori* sample size calculation was not feasible due to the lack of pre-existing reference effect sizes in the literature. Nevertheless, the final enrolled sample size of 680 total patients (and 320 undergoing fresh transfer) provides adequate degrees of freedom to fulfill the ‘events per variable’ criteria required for robust multivariable regression and double robust estimations.

## Results

### Patient characteristics

**Figure 1:**
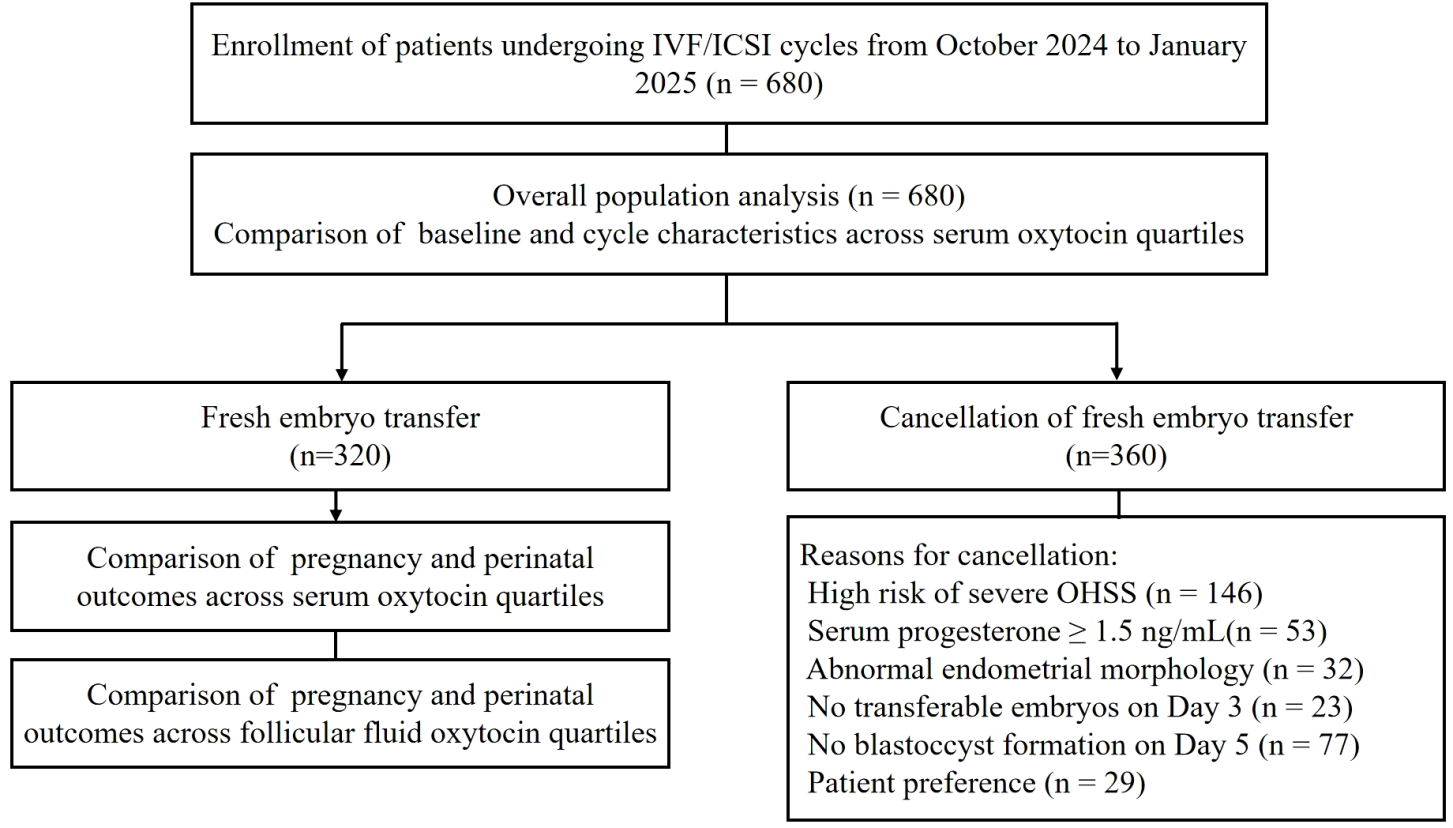
Flow diagram illustrating patient enrollment and analytic populations.

A total of 680 patients undergoing IVF/ICSI were stratified into quartiles based on their serum oxytocin levels. Of these enrolled patients, 320 (47.1%) underwent fresh embryo transfer, with no intergroup differences in transfer proportion (*P* = 0.410; **Supplementary Table 4**). Conversely, fresh transfers were canceled in 360 patients (52.9%) per our predefined freeze-all strategy (**Figure 1**). The primary indications for cancellation included a high risk of severe OHSS (n = 146), serum progesterone ≥1.5 ng/mL (n = 53), abnormal endometrial morphology (n = 32), lack of transferable embryos (n = 23), failure of blastocyst formation by Day 5 (n = 77), and patient preference (n = 29).

While baseline characteristics were largely comparable across groups (**Table 1**), serum oxytocin levels exhibited significant positive correlations with triglycerides, total cholesterol, LDL-C, and fasting insulin, alongside a negative correlation with HDL-C (all *P* < 0.01; **Supplementary Table 3**).

**Table 1:** Baseline characteristics stratified by serum oxytocin levels.

Parameters	Serum oxytocin levels stratified by quartiles (pg/mL)				
	< 25th (n=168)	25-50th (n=171)	51-75th (n=171)	> 75th (n=170)	*P* value
Age (years)	33 (30, 36)	33 (29, 37)	32 (29, 36)	33 (29, 37)	0.998
BMI (kg/m2)	21.6 (20.0, 23.7)	21.9 (20.3, 23.5)	21.2 (20.0, 23.2)	21.7 (20.6, 23.7)	0.637
Type of infertility					0.773
Primary	81 (48.2)	83 (48.5)	89 (52.0)	79 (46.5)	
Secondary	87 (51.8)	88 (51.5)	82 (48.0)	91 (53.5)	
Duration of infertility (years)	3 (2, 4)	3 (2, 5)	3 (2, 5)	3 (1, 5)	0.218
Causes of infertility					0.984
Tubal factor	70 (41.7)	78 (45.6)	64 (37.4)	73(42.9)	
Male factor	30 (17.9)	29 (17.0)	26 (15.2)	26 (15.3)	
Ovulation disorder DOR	11 (6.5) 19 (11.3)	9 (5.3) 13 (7.6)	11 (6.4) 19 (11.1)	11 (6.5) 19 (11.2)	
Other	10 (6.0)	7 (4.1)	12 (7.0)	9 (5.3)	
More than one etiology	16 (9.5)	20 (11.7)	25 (14.6)	20 (11.8)	
Unexplained	12 (7.1)	15 (8.8)	14 (8.2)	12 (7.1)	
AMH (ng/ml)	3.18 (1.56, 5.39)	3.09 (1.82, 5.95)	3.44 (1.63, 6.33)	3.42 (1.48, 5.70)	0.796
AFC	13.5 (8, 20)	14 (8, 19)	15 (8, 21)	12.5 (7.8, 22)	0.658
Basal FSH (IU/L)	7.01 (5.88, 8.34)	6.87 (5.89, 8.34)	6.53 (5.46, 8.08)	7.07 (5.88, 8.44)	0.364
Basal LH (IU/L)	5.74 (4.21, 7.20)	4.89 (3.84, 7.51)	5.12 (3.85, 6.94)	4.99 (3.84, 7.89)	0.454
Triglyceride (mmol/L)	1.11 (0.71, 1.57)^a^	1.09 (0.72, 1.62)^b^	1.35 (0.97, 1.88)^c^	1.56 (1.05, 2.52)^d^	**< 0.001**
Total cholesterol (mmol/L)	5.15 (4.28, 5.68)^a,b^	5.03 (4.19, 5.68)^a^	5.13 (4.26, 5.61)^a^	5.30 (4.66, 6.31)^b^	**< 0.001**
LDL-C (mmol/L)	3.08 (2.39, 3.54)^a^	2.95 (2.23, 3.42)^a^	3.17 (2.55, 3.53)^a^	3.34 (2.92, 3.83)^b^	**< 0.001**
HDL-C (mmol/L)	1.54 (1.22, 1.74)^a^	1.41 (1.18, 1.72)^a^	1.35 (1.12, 1.76)^a,b^	1.27 (1.13, 1.61)^b^	**< 0.001**
Fasting glucose (mmol/L)	4.88 (4.73, 5.06)	4.91 (4.68, 5.10)	4.91 (4.71, 5.20)	4.90 (4.73, 5.17)	0.522
Fasting insulin (μU/ml)	7.55 (5.80, 9.48)^a^	8.30 (6.10, 10.50)^a,b^	8.60 (6.80, 12.00)^b,c^	9.60 (7.60, 11.95)^c^	**< 0.001**

Data are presented as median (Q1, Q3) or number (percentage).

^a, b, c, d^ Different superscripts within the same line indicate statistical differences between subgroups. The quartiles of serum oxytocin levels were 130.1 pg/mL (25th percentile), 175.4 pg/mL (50th percentile), and 241.3 pg/mL (75th percentile). BMI, body mass index; DOR, diminished ovarian reserve; AMH, anti-Mullerian hormone; AFC, antral follicle count; LDL-C, low density lipoprotein-cholesterol; HDL-C, high density lipoprotein-cholesterol.

Bold indicates statistically significant P-values (P < 0.05).

### Cycle characteristics and ovarian response

Cycle characteristics and ovarian response parameters stratified by serum oxytocin quartiles are summarized in **Supplementary Table 4**. Most variables were comparable across groups, with no significant differences observed in gonadotropin dose, stimulation duration, serum estradiol, LH, or progesterone on hCG trigger day, ICSI proportion, or OHSS incidence. Although a marginal downward trend in 2PN, cleavage, and blastocyst formation rates was observed in the highest quartile (>75th percentile), overall intergroup differences did not reach statistical significance. Furthermore, variables among fresh transfer cycles—such as endometrial thickness, the number of embryos transferred, and the proportion of blastocyst transfers—remained comparable across groups. Additionally, serum and follicular fluid oxytocin levels showed no significant correlation (**Supplementary Table 3**; **Supplementary Figure 1**), and follicular fluid oxytocin concentrations did not differ across systemic serum quartiles (*P* = 0.274; **Supplementary Table 4**).

### Pregnancy and perinatal outcomes

**Figure 2:**
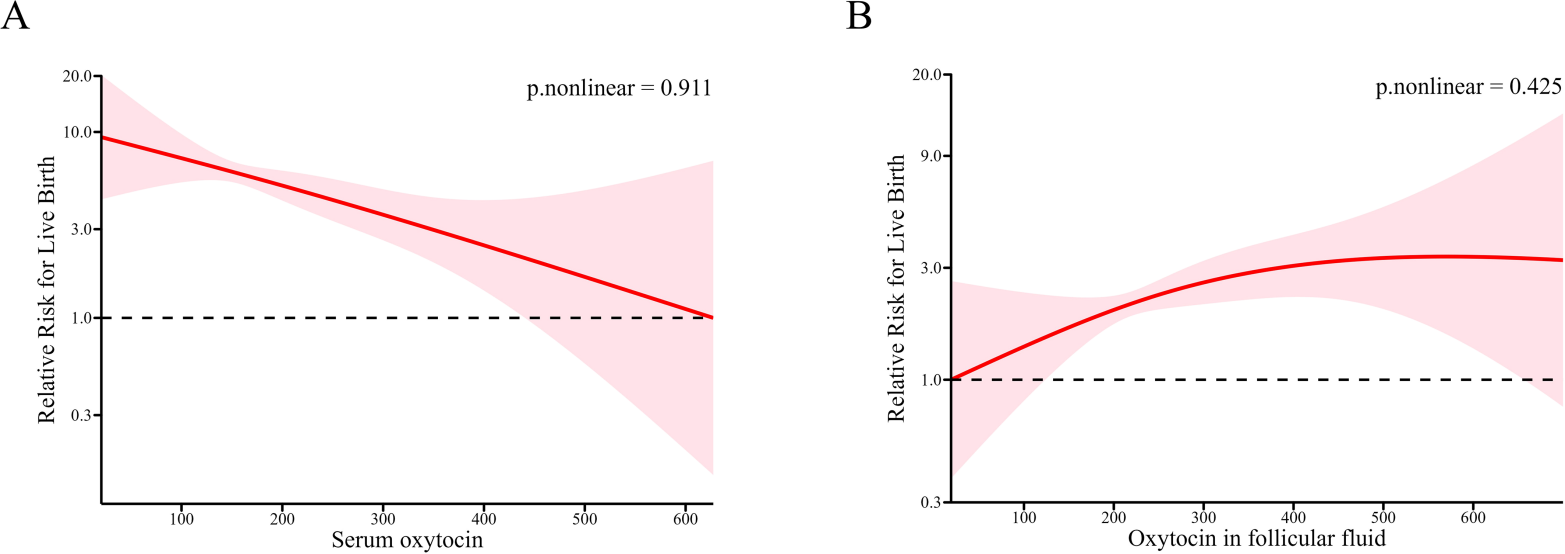
Smooth curve fitting models evaluating the associations between serum and follicular fluid oxytocin levels and live birth rate. **(A)** Smooth curve fitting of the association between serum oxytocin levels and the relative risk of live birth. **(B)** Smooth curve fitting of the association between follicular fluid oxytocin levels and the relative risk of live birth. Models were adjusted for age, BMI, AMH, causes of infertility, fasting insulin, days of stimulation, endometrial thickness, and serum estradiol and progesterone levels on the day of hCG trigger, and the number of embryos transferred. *p*.nonlinear indicates the *P-*value for nonlinear test.

Pregnancy and perinatal outcomes stratified by serum oxytocin quartiles are shown in **Table 2**. LBR differed significantly across groups (57.1%, 61.8%, 47.3%, and 37.2%; *P* < 0.001), with the highest quartile exhibiting a markedly lower LBR than the <25th and 25th–50th percentile groups. Implantation and clinical pregnancy rates were also significantly reduced in the highest quartile, whereas pregnancy loss and perinatal outcomes remained comparable among groups. Adjusted smooth curve fitting revealed a linear negative association between serum oxytocin levels and live birth (*P* for non-linearity = 0.911; **Figure 2A**).

**Table 2:** Pregnancy and perinatal outcomes of fresh embryo transfer stratified by serum oxytocin levels.

Parameters	Serum oxytocin levels stratified by quartiles				
	< 25th (n=84)	25-50th (n=76)	51-75th (n=74)	> 75th (n=86)	*P* value
Live birth	48 (57.1)^a^	47 (61.8)^a^	35 (47.3)^a,b^	32 (37.2)^b^	**0.008**
Implantation rate	70/130 (53.8)^a^	53/116 (45.7)^a,b^	51/118 (43.2)^a,b^	46/132 (34.8)^b^	**0.021**
Biochemical pregnancy	63 (75.0)^a^	53 (69.7)^a,b^	48 (64.9)^a,b^	48 (55.8)^b^	0.056
Clinical pregnancy	57 (67.9)^a^	51 (67.1)^a^	40 (54.1)^a,b^	38 (44.2)^b^	**0.004**
Pregnancy loss	7/57 (12.3)	4/51 (7.8)	4/40 (10.0)	5/38 (13.2)	0.840
Singleton	42/48 (87.5)	43/47 (91.5)	30/35 (85.7)	28/32 (87.5)	0.864
Newborn sex*					0.951
Female	22 (52.4)	22 (51.2)	15 (50.0)	16 (57.1)	
Male	20 (47.6)	21 (48.8)	15 (50.0)	12 (42.9)	
Gestational age*	38 (37, 39)	38 (38, 39)	38.5 (37, 40)	38 (37, 39)	0.378
Mode of delivery*					0.610
Vaginal	26/42 (61.9)	25/43 (58.1)	20/30 (66.7)	14/28 (50.0)	
Cesarean section	16/42 (38.1)	18/43 (41.9)	10/30 (33.3)	14/28 (50.0)	
Preterm birth*	5/42 (11.9)	4/43 (9.3)	2/30 (6.7)	5/28 (17.9)	0.563
Low birthweight*	2/42 (4.8)	3/43 (7.0)	2/30 (6.7)	2/28 (7.1)	0.970
High birthweight*	0	2/43 (4.7)	0	0	0.194
Birthweight*	3130 (2933, 3310)	3200 (2930, 3450)	3225 (2785, 3500)	3165 (2978, 3310)	0.527
Z-score*	-0.17 (-0.52, 0.42)	0.16 (-0.61, 0.64)	0.09 (-0.94, 0.85)	-0.18 (-0.43, 0.29)	0.892
Small for gestational age*	2/42 (4.8)	3/43 (7.0)	3/30 (10.0)	1/28 (3.6)	0.741
Large for gestational age*	1/42 (2.4)	4/43 (9.3)	1/30 (3.3)	1/28 (3.6)	0.453

Data are presented as median (Q1, Q3) or number (percentage). *Among all singletons. ^a, b^Different superscripts within the same line indicate statistical differences between subgroups. The quartiles of serum oxytocin levels were 130.1 pg/mL (25th percentile), 175.4 pg/mL (50th percentile), and 241.3 pg/mL (75th percentile).

Bold indicates statistically significant P-values (P < 0.05).

Three statistical models were constructed to further evaluate this association (**Table 3**). Multivariable logistic regression (Model 1) and inverse probability weighting (Model 2) consistently demonstrated that elevated serum oxytocin significantly decreased the likelihood of live birth, whereas follicular fluid oxytocin showed a positive trend. Ultimately, the double robust estimation (Model 3) confirmed these fully adjusted stage-specific effects: each 100 pg/mL increase in serum oxytocin was linked to a 37% reduced likelihood of live birth (95% CI: 25%–47%), contrasted by an 18% increase associated with follicular fluid oxytocin (95% CI: 2%–37%).

**Table 3:** Association between serum and follicular oxytocin levels and live birth rate estimated using three different models.

Variables	Model 1			Model 2			Model 3		
	Adjusted OR	95% CI	*P* value	Adjusted OR	95% CI	*P* value	Adjusted OR	95% CI	*P* value
Age	0.88	0.83-0.94	< 0.001	0.89	0.85-0.93	< 0.001	0.90	0.86-0.94	< 0.001
BMI (kg/m2)	1.02	0.96-1.08	0.512	1.02	0.98-1.06	0.393	1.01	0.97-1.06	0.518
Causes of infertility									
Tubal factor	Reference			Reference			Reference		
Male factor	1.27	0.64-2.50	0.497	1.26	0.79-2.02	0.332	1.60	0.99-2.60	0.057
Ovulation disorder	1.13	0.35-3.68	0.841	0.55	0.24-1.25	0.152	0.24	0.10-0.56	**0.001**
DOR	0.79	0.31-2.00	0.620	0.84	0.46-1.53	0.572	0.94	0.52-1.72	0.846
Other	0.20	0.03-1.23	0.082	0.13	0.04-0.42	**< 0.001**	0.14	0.04-0.42	**< 0.001**
More than one etiology	2.20	0.90-5.40	0.084	2.88	1.59-5.23	**< 0.001**	2.59	1.47-4.54	**0.001**
Unexplained	1.77	0.72-4.38	0.215	1.59	0.84-3.02	0.154	1.78	0.91-3.50	0.091
AMH (ng/ml)	1.02	0.95-1.11	0.552	1.04	0.99-1.10	0.127	1.10	1.04-1.16	**0.002**
Serum oxytocin (per 100pg/ml increment)	0.70	0.54-0.90	**0.007**	0.65	0.54-0.79	**< 0.001**	0.63	0.53-0.75	**< 0.001**
Fasting insulin (μU/ml)	1.01	0.96-1.07	0.626	1.02	0.98-1.06	0.270	1.03	0.99-1.07	0.105
Days of stimulation	1.11	0.97-1.28	0.133	1.13	1.03-1.25	**0.014**	1.18	1.07-1.31	**0.001**
Endometrial thickness*	1.06	0.96-1.17	0.273	1.07	0.99-1.16	0.076	1.07	0.99-1.16	0.076
Estradiol on hCG trigger day (pg/mL)	1.00	1.00-1.00	0.709	1.00	1.00-1.00	0.368	1.00	1.00-1.00	0.345
Progesterone on hCG trigger day (ng/mL)	0.70	0.37-1.32	0.267	1.05	0.77-1.44	0.766	1.08	0.80-1.45	0.627
Follicular oxytocin (per 100pg/ml increment)	1.21	0.98-1.49	0.071	1.23	1.07-1.42	**0.005**	1.18	1.02-1.37	**0.023**
No. of embryos transferred (2 vs. 1)	1.71	1.01-2.90	**0.046**	1.71	1.18-2.47	**0.005**	1.71	1.17-2.50	**0.006**

OR, odds ratio; CI, confidence interval; BMI, body mass index; DOR, diminished ovarian reserve; AMH, anti-Mullerian Hormone.

Model 1, Multivariable regression model; Model 2, Inverse probability weighting (IPW); Model 3, Double robust model (DRM).

Bold indicates statistically significant P-values (P < 0.05).

### Clinical outcomes stratified by follicular fluid oxytocin levels

Spearman correlation analysis indicated that follicular fluid oxytocin levels were positively associated with markers of ovarian response—including AMH, AFC, stimulation duration, estradiol/progesterone on hCG trigger day, and numbers of oocyte retrieved—and negatively associated with age, basal FSH, and LH on trigger day (**Supplementary Table 3**). Cycle characteristics stratified by follicular fluid oxytocin quartiles aligned with these correlations (**Supplementary Table 5**). The lowest quartile (<25th percentile) exhibited lower estradiol and progesterone on trigger day, alongside fewer retrieved oocytes and cleavage embryos, while the highest quartile (>75th percentile) had a lower proportion of fresh transfers.

Among patients undergoing fresh transfer, LBR increased significantly across follicular oxytocin quartiles (45.5%, 40.4%, 59.1%, and 60.7%; *P* = 0.021), reflecting a linear positive trend confirmed by smoothed curve fitting (*P* for non-linearity = 0.425; **Supplementary Table 6**; **Figure 2B**). Implantation, biochemical, and clinical pregnancy rates followed a similar pattern, though mean birthweight was slightly lower in the highest quartile (*P* = 0.038).

## Discussion

This study demonstrates that serum oxytocin levels prior to ovarian stimulation are negatively associated with live birth following fresh embryo transfer, whereas follicular fluid oxytocin levels are positively associated with ovarian response and live birth rate.

Oxytocin is best known for regulating uterine contractions during labor. Its antagonist, atosiban, has been used to prevent preterm labor and, more recently, around embryo transfer; however, evidence for improved ART outcomes remains inconsistent (10). The role of endogenous oxytocin in IVF outcomes is poorly understood, and no prior studies have examined follicular fluid oxytocin. Only one previous study evaluated serum oxytocin at multiple time points in patients with RIF, prior successful transfer, or failed transfer (11). That study included only 108 patients across three groups (n = 38, 41, and 39), limiting statistical power. Although no significant differences were observed among groups, serum oxytocin showed an upward trend in the RIF group. In addition, samples were collected after GnRH agonist downregulation, which may have influenced oxytocin levels.

Excessive uterine contractions can impair implantation by expelling embryos from the uterine cavity (9, 17). In animal models, oxytocin has been shown to reduce implantation rates—an effect reversed by oxytocin antagonists (18). In our prospective cohort, serum oxytocin measured during menstruation before ovarian stimulation showed a negative linear association with live birth rate after fresh embryo transfer, suggesting that elevated basal oxytocin may adversely modulate the maternal environment and/or uterine contractility. Few studies have focused on endogenous oxytocin levels in women. One study reported significantly higher plasma oxytocin levels in women with dysmenorrhea compared to healthy controls during menstruation, suggesting that basal oxytocin may contribute to primary dysmenorrhea by promoting uterine contractions (19).

In contrast, follicular fluid oxytocin levels were not correlated with serum oxytocin levels in our study, likely reflecting local ovarian synthesis and differences in sampling timing. Oxytocin production in the ovary increases throughout the follicular phase in parallel with rising estrogen levels (4, 5). Consistently, follicular oxytocin levels in our study were positively correlated with estradiol on the hCG trigger day and with oocyte yield. Although serum oxytocin was not measured at the time of oocyte retrieval, we speculate that serum levels at that time may correlate positively with follicular concentrations. Xia et al. reported that in stimulation cycles, serum oxytocin levels were significantly higher around oocyte retrieval than before stimulation (11), possibly contributing to increased uterine contractility and adverse pregnancy outcomes (20, 21).

Notably, follicular oxytocin levels in our study were positively associated with live birth rate, suggesting that elevated follicular oxytocin may reflect a stronger ovarian response and improved pregnancy outcomes. Supporting this hypothesis, previous research has shown that oxytocin can promote the LH surge (22), whereas inhibition by atosiban can disrupt the ovulatory process (23). These findings may help explain why previous studies failed to show consistent associations between oxytocin levels and IVF outcomes: oxytocin around the time of oocyte retrieval may reflect both uterine contractility and ovarian response, whose opposing effects may offset each other. These dual and opposing effects may also contribute to the inconsistent findings observed with oxytocin antagonists in IVF cycles (10).

Our findings reveal an apparent paradox: elevated baseline serum oxytocin is associated with lower LBR, whereas higher follicular fluid oxytocin correlates with improved LBR. This dichotomy can be explained by the distinct temporal and spatial dynamics of systemic versus local oxytocin. The negative association between basal systemic oxytocin and live birth may be driven by both direct mechanical and indirect metabolic mechanisms. Directly, elevated systemic oxytocin during the menstrual phase may predispose the uterus to hypercontractility and mechanically impair implantation (9, 17). Indirectly, our correlation analysis revealed significant positive associations between serum oxytocin and metabolic markers—specifically triglycerides, total cholesterol, LDL-C, and fasting insulin—alongside a negative correlation with HDL-C. These findings align with previous reports linking elevated oxytocin to dyslipidemia and insulin resistance in non-diabetic populations (24, 25). The lack of correlation with BMI likely reflects our cohort’s relatively young age, low mean BMI, and the exclusion of diabetic individuals. We speculate that elevated basal oxytocin in this specific demographic may act as an early physiological correlate of subclinical metabolic dysregulation (26, 27). This underlying metabolically suboptimal environment is well-documented to impair endometrial receptivity, potentially by downregulating critical implantation markers such as integrins and leukemia inhibitory factor (28, 29). Thus, high basal serum oxytocin acts as a systemic proxy for a suboptimal maternal environment leading to adverse IVF outcomes. However, since our observational data cannot confirm these mechanistic links, future basic and translational studies are required to validate these proposed metabolic interactions.

This is the first prospective cohort study to simultaneously evaluate baseline serum and follicular fluid oxytocin levels in IVF patients and to examine their associations with IVF outcomes. Serum oxytocin was measured during menstruation prior to ovarian stimulation, reflecting baseline physiological oxytocin status without the confounding effects of supraphysiological hormonal exposure during stimulation. In parallel, follicular fluid oxytocin was assessed, providing novel insight into the potential role of locally produced oxytocin in ovarian response. In addition, we applied multiple complementary analytical approaches—including multivariable regression, inverse probability weighting, and a double robust model—to address potential confounding and selection bias. The consistency of results across these models supports the robustness of our findings.

Several limitations should be acknowledged. First, serum oxytocin was measured only once during the menstrual phase prior to ovarian stimulation. Given the known dynamic fluctuations of endogenous oxytocin across the menstrual cycle and in response to exogenous gonadotropin stimulation, a single baseline measurement may not accurately reflect peri-transfer or peri-implantation systemic levels. Consequently, the lack of correlation between serum and follicular fluid oxytocin likely reflects this difference in sampling timing, underscoring the need for future studies to assess serum oxytocin at multiple time points throughout the IVF cycle. Second, only 320 patients underwent fresh embryo transfer, raising the possibility of selection bias when evaluating live birth outcomes. Although inverse probability weighting and a double robust model were used to mitigate this bias and yielded consistent results, residual bias cannot be entirely excluded. Finally, the observational nature of the study precludes causal inference.

Despite these methodological constraints, our dual-compartment findings provide preliminary insights into precision ART strategies. First, regarding systemic profiles, while routine screening of basal serum oxytocin is not currently warranted, identifying subclinically elevated levels could theoretically help inform the highly debated utility of oxytocin antagonists (e.g., atosiban) during embryo transfer. Rather than generalized empirical usage, targeted antagonist application might be considered for subpopulations with high baseline systemic oxytocin, potentially mitigating an unfavorable hypercontractile environment. Second, the positive correlation between follicular fluid oxytocin and oocyte yield suggests its potential value as a correlate of ovarian response. Evaluating these local pathways may eventually aid in assessing follicular maturation. Ultimately, to bridge the gap between these conceptual models and clinical utility, future longitudinal tracking and prospectively stratified randomized trials are essential to validate these targeted therapeutic benefits.

Our findings demonstrate that elevated basal oxytocin levels are negatively associated with live birth rates following fresh embryo transfer, whereas higher follicular fluid oxytocin concentrations correlate positively with ovarian response and pregnancy outcomes. Although these findings underscore the dual roles of oxytocin in reproductive physiology, future longitudinal studies tracking its dynamics across IVF cycles are required to establish its clinical predictive utility and to explore targeted therapeutic interventions.

## Data Availability

The raw data supporting the conclusions of this article will be made available by the authors, without undue reservation.
