# Supplementary material for: Association between endogenous oxytocin levels and live birth rates following fresh embryo transfer: a prospective cohort study
Source: Front Endocrinol (Lausanne). 2026 Jun 2;17:1850346. doi: 10.3389/fendo.2026.1850346 (PMC13268967; doi:10.3389/fendo.2026.1850346)
Supplement: Supplementary file 1 [file DataSheet1.docx]

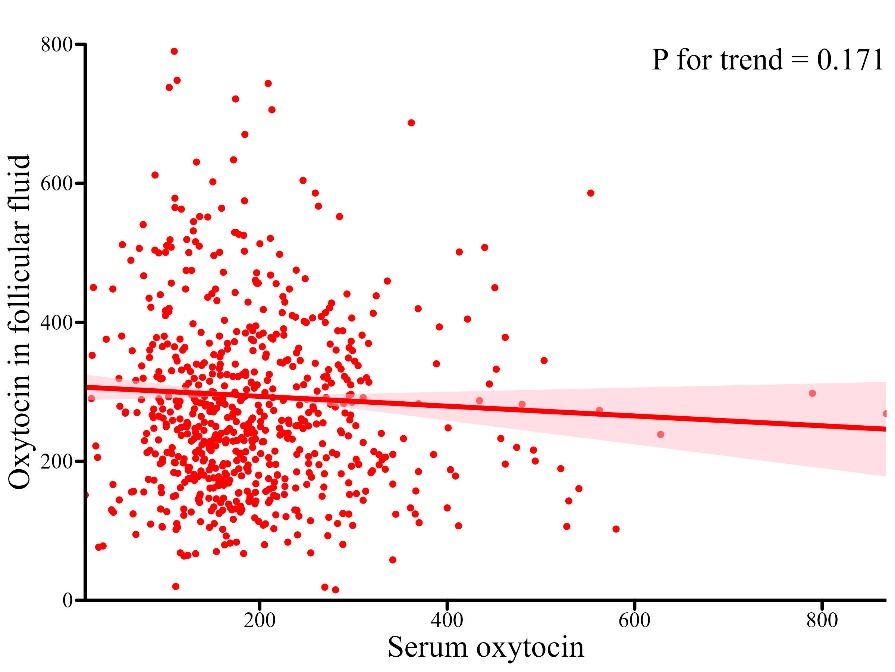


**Figure S1.** Association between serum oxytocin and follicular fluid oxytocin concentrations. A smoothed curve-fitting model was constructed to evaluate the association between serum and follicular fluid oxytocin levels (pg/ml).
