# Supplementary material for: Association between endogenous oxytocin levels and live birth rates following fresh embryo transfer: a prospective cohort study
Source: Front Endocrinol (Lausanne). 2026 Jun 2;17:1850346. doi: 10.3389/fendo.2026.1850346 (PMC13268967; doi:10.3389/fendo.2026.1850346)
Supplement: Supplementary file 2 [file DataSheet2.docx]

**Table S1** Comparisons of baseline and cycle characteristics according whether patients achieved live birth after fresh embryo transfer.

| **Parameters** | **No live birth (n = 158)** | **Live birth (n = 162)** | ***P* value** |
| --- | --- | --- | --- |
| Age (years) | 34 (30, 37) | 32 (29, 35) | **< 0.001** |
| BMI (kg/m^2^)  Type of infertility  Primary  Secondary  Duration of infertility  Causes of infertility  Tubal factor  Male factor  Ovulation disorder  DOR  Other  More than one etiology  Unexplained  Serum oxytocin (pg/mL)  AMH (ng/ml) | 21.5 (20.1, 23.4)  72 (45.6)  86 (54.4)  3 (2, 5)  80 (50.6)  21 (13.3)  8 (5.1)  19 (12.0)  7 (4.4)  13 (8.2)  10 (6.3)  189.5 (131.6, 267.8)  2.32 (1.21, 4.80) | 21.2 (19.6, 23.9)  79 (48.8)  83 (51.2)  3 (2, 4)  73 (45.1)  31 (19.1)  8 (4.9)  10 (6.2)  2 (1.2)  20 (12.3)  18 (11.1)  157.8 (117.5, 222.5)  3.32 (1.86, 5.30) | 0.845  0.567  0.310  0.073  **0.003**  **0.004** |
| AFC  Basal FSH (IU/L)  Basal LH (IU/L)  Triglyceride (mmol/L)  Total cholesterol (mmol/L)  LDL-C (mmol/L)  HDL-C (mmol/L)  Fasting glucose (mmol/L)  Fasting insulin (μU/ml)  Gonadotropin dose  Days of stimulation  Estradiol on hCG trigger day (pg/mL)  LH on hCG trigger day (IU/L)  Progesterone on hCG trigger day (ng/mL)  Endometrial thickness (mm)*  Oxytocin in follicular fluid  ICSI treatment | 11.0 (8.0, 17.3)  7.31 (5.87, 8.73)  4.76 (3.89, 7.35)  1.12 (0.79, 1.79)  5.16 (4.27, 5.74)  3.12 (2.44, 3.54)  1.39 (1.19, 1.71)  4.89 (4.69, 5.09)  8.50 (6.78, 10.43)  2250 (1563, 2700)  10 (9, 11.3)  2223 (1408, 3342)  2.45 (1.63, 4.15)  0.81 (0.52, 1.03)  11 (10, 13)  232.4 (180.2, 301.4)  38 (24.1) | 14.0 (9.8, 20.3)  6.77 (5.90, 8.15)  5.28 (3.92, 6.86)  1.35 (0.74, 1.87)  5.13 (4.29, 5.75)  3.09 (2.49, 3.81)  1.36 (1.15, 1.71)  4.91 (4.73, 5.19)  8.10 (6.00, 10.23)  2025 (1419, 2700)  10.5 (9, 12)  2492 (1749, 3456)  2.25 (1.45, 3.90)  0.72 (0.45, 0.92)  11 (10, 13.3)  268.1 (197.4, 336.9)  46 (28.4) | **0.001**  0.075  0.816  0.840  0.811  0.554  0.510  0.429  0.277  0.174  **0.013**  **0.043**  0.215  **0.046**  **0.010**  **0.010**  0.377 |
| No. of embryos transferred |  |  | 0.271 |
| Single | 76 (48.1) | 68 (42.0) |  |
| Double  Blastocyst transfer  Route of progesterone supplementation  Intramuscular  Vaginal + oral | 82 (51.9)  68 (43.0)  56 (35.4)  102 (64.6) | 94 (58.0)  63 (38.9)  54 (33.3)  108 (66.7) | 0.450  0.691 |

Data are presented as median (Q1, Q3) or number (percentage). BMI, body mass index; DOR, diminished ovarian reserve; AMH, anti-Mullerian hormone; AFC, antral follicle count; LDL-C, low density lipoprotein-cholesterol; HDL-C, high density lipoprotein-cholesterol.

**Table S2** Comparisons of baseline and cycle characteristics according to whether patients underwent fresh embryo transfer.

| **Parameters** | **No ET (n = 360)** | **ET (n = 320)** | ***P* value** |
| --- | --- | --- | --- |
| Age | 33 (29, 37) | 33 (30, 36) | 0.851 |
| BMI (kg/m^2^)  Type of infertility  Primary  Secondary  Duration of infertility  Causes of infertility  Tubal factor  Male factor  Ovulation disorder  DOR  Other  More than one etiology  Unexplained  Serum oxytocin (pg/mL)  AMH (ng/ml) | 21.8 (20.3, 23.6)  181 (50.3)  179 (49.7)  3 (2, 5)  132 (36.7)  59 (16.4)  26 (7.2)  41 (11.4)  29 (8.1)  48 (13.3)  25 (6.9)  175.8 (132.9, 239.1)  3.74 (1.72, 7.02) | 21.4 (20.0, 23.5)  151 (47.2)  169 (52.8)  3 (2, 5)  153 (47.8)  52 (16.3)  16 (5.0)  29 (9.1)  9 (2.8)  33 (10.3)  28 (8.8)  175.3 (123.5, 247.7)  2.81 (1.64, 5.16) | 0.176  0.421  0.552  **0.007**  0.691  **0.001** |
| AFC  Basal FSH (IU/L)  Basal LH (IU/L)  Triglyceride (mmol/L)  Total cholesterol (mmol/L)  LDL-C (mmol/L)  HDL-C (mmol/L)  Fasting glucose (mmol/L)  Fasting insulin (μU/ml)  Gonadotropin dose  Days of stimulation  Estradiol on hCG trigger day (pg/mL)  LH on hCG trigger day (IU/L)  Progesterone on hCG trigger day (ng/mL)  Endometrial thickness (mm)* | 15 (8, 22)  6.78 (5.64, 8.11)  5.39 (3.91, 7.92)  1.35 (0.89, 1.93)  5.15 (4.34, 5.74)  3.15 (2.59, 3.53)  1.41 (1.16, 1.71)  4.90 (4.72, 5.12)  8.60 (6.63, 11.60)  1800 (1200, 2500)  10 (9, 11)  3281 (1552, 5161)  2.37 (1.49, 4.59)  0.88 (0.55, 1.26)  11 (10, 13) | 13 (9, 19)  7.02 (5.90, 8.42)  4.92 (3.90, 7.04)  1.25 (0.77, 1.87)  5.14 (4.28, 5.74)  3.11 (2.45, 3.70)  1.38 (1.16, 1.71)  4.90 (4.71, 5.11)  8.30 (6.40, 10.30)  2088 (1500, 2700)  10 (9, 12)  2296 (1499, 3358)  2.28 (1.54, 4.09)  0.73 (0.48, 0.95)  11 (10, 13) | 0.065  0.267  0.112  0.231  0.673  0.978  0.550  0.990  0.163  **0.001**  0.052  **< 0.001**  0.626  **< 0.001**  0.106 |
| Moderate or severe OHSS rate | 23 (6.4) | 18 (5.6) | 0.676 |

Data are presented as median (Q1, Q3) or number (percentage). ET, embryo transfer; BMI, body mass index; DOR, diminished ovarian reserve; AMH, anti-Mullerian hormone; AFC, antral follicle count; LDL-C, low density lipoprotein-cholesterol; HDL-C, high density lipoprotein-cholesterol; OHSS, ovarian hyperstimulation syndrome.

**Table S3** Correlation analyses of serum and follicular oxytocin with clinical variables.

|  | **Serum oxytocin**  **Spearman correlation** | | **Follicular oxytocin**  **Spearman correlation** | |
| --- | --- | --- | --- | --- |
| **Parameters** | ***r*** | ***P*** | ***r*** | ***P*** |
| Age | -0.006 | 0.875 | -0.099 | **0.010** |
| BMI | -0.016 | 0.669 | 0.072 | 0.059 |
| Duration of infertility | 0.054 | 0.157 | -0.045 | 0.242 |
| AMH | 0.022 | 0.568 | 0.183 | **< 0.001** |
| AFC  Basal FSH | 0.016  -0.018 | 0.684  0.640 | 0.195  -0.198 | **< 0.001**  **< 0.001** |
| Basal LH  Triglyceride  Total cholesterol  LDL-C  HDL-C  Fasting glucose  Fasting insulin  Gonadotropin dose  Days of stimulation  Estradiol on hCG trigger day  LH on hCG trigger day  Progesterone on hCG trigger day  No. of oocytes retrieved  Endometrial thickness (mm)  Oxytocin in follicular fluid | -0.038  0.294  0.094  0.189  -0.167  0.049  0.257  -0.030  -0.013  0.005  -0.012  0.026  0.028  0.010  -0.036 | 0.321  **< 0.001**  **0.015**  **< 0.001**  **< 0.001**  0.199  **< 0.001**  0.428  0.741  0.903  0.770  0.513  0.472  0.865  0.354 | -0.015  -0.027  0.029  0.018  -0.018  0.043  0.022  0.019  0.160  0.229  -0.211  0.182  0.248  0.062 | 0.690  0.486  0.452  0.642  0.644  0.266  0.568  0.614  **< 0.001**  **< 0.001**  **< 0.001**  **< 0.001**  **< 0.001**  0.267 |

BMI, body mass index; AMH, anti-Mullerian hormone; AFC, antral follicle count; LDL-C, low density lipoprotein-cholesterol; HDL-C, high density lipoprotein-cholesterol.

**Table S4** Cycle characteristics and ovarian response outcomes stratified by serum oxytocin levels.

| **Parameters** | **Serum oxytocin levels stratified by quartiles** | | | | | |
| --- | --- | --- | --- | --- | --- | --- |
|  | **< 25th (n=168)** | **25-50th (n=171)** | **51-75th (n=171)** | **> 75th (n=170)** | ***P* value** |  |
| Gonadotropin dose  Days of stimulation  Estradiol on hCG trigger day  LH on hCG trigger day  Progesterone on hCG trigger day  Oxytocin in follicular fluid  ICSI treatment  No. of oocytes retrieved  No. of 2PN  Rate of 2PN  No. of cleavage embryos on day 3  Rate of cleavage embryos on day 3  No. of blastocysts  Rate of blastocysts  Moderate or severe OHSS rate  Fresh embryo transfer cycles  Endometrial thickness (mm)*  No. of embryos transferred^#^ | 2038 (1500, 2719)  10 (9, 12)  2740 (1485, 4352)  2.25 (1.46, 4.25)  0.76 (0.52, 1.07)  270.9 (191.2, 375.8)  57 (33.9)  13 (7, 20)  8 (4, 12)  1426/2186 (65.2)  7 (3, 11)  1258/1726 (72.9)  4 (1, 7)  496/1178 (42.1)  12 (7.1)  84/168 (50.0)  11 (10, 13) | 2000 (1350, 2550)  10 (9, 12)  2654 (1487, 4430)  2.72 (1.58, 4.98)  0.79 (0.50, 1.14)  242.9 (177.8, 322.1)  55 (32.2)  12 (6, 20)  8 (4, 12)  1478/2296 (64.4)  7 (3, 11)  1280/1807 (70.8)  4 (2, 7)  539/1211 (44.5)  7 (4.1)  76/171 (44.4)  11 (10, 13) | 1800 (1200, 2625)  10 (9, 11)  2636 (1554, 4386)  2.25 (1.45, 4.39)  0.80 (0.53, 1.08)  251.0 (180.6, 354.2)  59 (34.5)  13 (8, 20)  8 (4, 12)  1464/2275 (64.4)  7 (3, 11)  1267/1805 (70.2)  4 (2, 7)  504/1165 (43.3)  14 (8.2)  74/171 (43.3)  11 (10, 13) | 2025 (1350, 2700)  10 (9, 12)  2628 (1573, 4328)  2.33 (1.49, 3.90)  0.84 (0.53, 1.18)  263.9 (188.4, 340.4)  58 (34.1)  13 (8, 20)  8 (4, 12)  1410/2284 (61.7)  7 (3, 11)  1199/1747 (68.6)  3 (2, 6)  477/1212 (39.4)  8 (4.7)  86/170 (50.6)  11 (10, 13) | 0.099  0.162  0.996  0.163  0.740  0.274  0.970  0.825  0.979  0.080  0.999  0.050  0.262  0.066  0.330  0.410  0.990  0.838 |  |
| Single | 38 (45.2) | 36 (47.4) | 30 (40.5) | 40 (46.5) |  |  |
| Double  Blastocyst transfer^#^ | 46 (54.8)  32 (38.1) | 40 (52.6)  34 (44.7) | 44 (59.5)  29 (39.2) | 46 (53.5)  36 (41.9) | 0.835 |  |
| Route of progesterone supplementation^#^  Intramuscular + Oral  Vaginal + Oral | 32 (38.1)  52 (61.9) | 24 (31.6)  52 (68.4) | 25 (33.8)  49 (66.2) | 29 (33.7)  57 (66.3) | 0.848 |  |

Data are presented as median (Q1, Q3) or number (percentage). *The day on hCG trigger day. ^#^Among fresh embryo transfer cycles.

The quartiles of serum oxytocin levels were 130.1 pg/mL (25th percentile), 175.4 pg/mL (50th percentile), and 241.3 (75th percentile). 2PN, 2 pronuclear; OHSS, ovarian hyperstimulation syndrome.

**Table S5** Cycle characteristics and ovarian response outcomes stratified by oxytocin levels in follicular fluid.

| **Parameters** | **Oxytocin levels in FF stratified by quartiles** | | | | | |
| --- | --- | --- | --- | --- | --- | --- |
|  | **< 25th (n=169)** | **25-50th (n=172)** | **51-75th (n=170)** | **> 75th (n=169)** | ***P* value** |  |
| Oxytocin in FF (pg/ml)  Gonadotropin dose  Days of stimulation  Estradiol on hCG trigger day  LH on hCG trigger day  Progesterone on hCG trigger day  ICSI treatment  No. of oocytes retrieved  No. of 2PN  Rate of 2PN  No. of cleavage embryos on day 3  Rate of cleavage embryos on day 3  No. of blastocysts  Rate of blastocysts  Fresh embryo transfer cycles  Endometrial thickness (mm)*  No. of embryos transferred^#^ | 142.9 (113.3, 165.2)^a^  2025 (1363, 2700)  10 (8, 11)^a^  1726 (725, 3497)^a^  3.26 (1.91, 5.92)^a^  0.61 (0.33, 0.94)^a^  51 (30.2)  7 (4, 18)^a^  4 (2, 9)^a^  1057/1713 (61.7)^a^  4 (2, 8)^a^  928/1339 (69.3)  4 (1, 6)  349/807 (43.2)  77/169 (45.6)^a,b^  10 (9, 12.5) | 222.2 (202.6, 241.1)^b^  1800 (1213, 2494)  10 (9, 11)^a,b^  2701 (1666, 4860)^b^  2.28 (1.53, 4.74)^b^  0.83 (0.59, 1.18)^b^  53 (30.8)  14 (8, 20)^b^  9 (5, 12)^b^  1581/2414 (65.5)^b^  8 (4, 11)^b^  1376/1938 (71.0)  4 (2, 6)  563/1310 (43.0)  94/172 (54.7)^b^  11 (10, 13) | 295.2 (278.9, 321.4)^c^  2025 (1400, 2700)  10 (9, 12)^b^  2745 (1829, 4386)^b^  2.25 (1.46, 4.25)^b,c^  0.84 (0.58, 1.09)^b^  58 (34.1)  13 (8, 20)^b^  8 (5, 12)^b^  1495/2373 (63.0)^a,b^  7 (4, 11)^b^  1301/1845 (70.5)  3 (2, 6)  533/1252 (42.6)  88/170 (51.8)^b^  11 (10, 14) | 434.8 (380.2, 510.5)^d^  1875 (1350, 2700)  10 (9, 12)^b^  3262 (2001, 4855)^b^  2.02 (1.34, 3.29)^c^  0.85 (0.60, 1.18)^b^  67 (39.6)  16 (10, 20)^b^  9 (6, 13)^b^  1645/2541 (64.7)^b^  8 (5, 11)^b^  1399/1963 (71.3)  4 (1, 7)  571/1397 (40.9)  61/169 (36.1)^a^  11 (10, 13) | **< 0.001**  0.265  **< 0.001**  **< 0.001**  **< 0.001**  **< 0.001**  0.234  **< 0.001**  **< 0.001**  **0.049**  **< 0.001**  0.646  0.702  0.627  **0.003**  0.118  0.979 |  |
| Single | 33 (42.9) | 43 (45.7) | 40 (45.5) | 28 (45.5) |  |  |
| Double  Blastocyst transfer^#^ | 44 (57.1)  30 (39.0) | 51 (54.3)  39 (41.5) | 48 (54.5)  36 (40.9) | 33 (54.1)  26 (42.6) | 0.976 |  |
| Route of progesterone supplementation^#^  Intramuscular + Oral  Vaginal + Oral  Moderate or severe OHSS rate | 32 (41.6)  45 (58.4)  7 (4.1) | 31 (33.0)  63 (67.0)  11 (6.4) | 26 (29.5)  62 (70.5)  10 (5.9) | 21 (34.4)  40 (65.6)  13 (7.7) | 0.431  0.586 |  |

Data are presented as median (Q1, Q3) or number (percentage). *The day on hCG trigger day. #Among fresh embryo transfer cycles.

^a, b,c^ Different superscripts within the same line indicate statistical differences between subgroups. The quartiles of follicular oxytocin levels were 183.8 pg/mL (25th percentile), 257.7 pg/mL (50th percentile), and 345.0 pg/mL (75th percentile). FF, follicular fluid; 2PN, 2 pronuclear; OHSS, ovarian hyperstimulation syndrome.

**Table S6** Pregnancy and perinatal outcomes of fresh embryo transfer stratified by oxytocin levels in follicular fluid.

| **Parameters** | **Oxytocin levels in FF stratified by quartiles** | | | | |
| --- | --- | --- | --- | --- | --- |
|  | **< 25th (n=77)** | **25-50th (n=94)** | **51-75th (n=88)** | **> 75th (n=61)** | ***P* value** |
| Live birth  Implantation rate  Biochemical pregnancy  Clinical pregnancy  Pregnancy loss  Singleton  Newborn sex*  Female  Male  Gestational age*  Mode of delivery*  Vaginal  Cesarean section  Preterm birth*  Low birthweight*  High birthweight*  Birthweight*  Z-score*  Small for gestational age*  Large for gestational age* | 35 (45.5)^a,b^  47/121 (38.8)^a,b^  44 (57.1)^a^  39 (50.6)^a,b^  3/39 (7.7)  30/35 (85.7)  16 (53.3)  14 (46.7)  38 (37, 39)  15/30 (50.0)  15/30 (50.0)  2/30 (6.7)  2/30 (6.7)  1/30 (3.3)  3260 (3100, 3503)^a^  0.21 (-0.38, 0.93)  1/30 (3.3)  3/30 (10.0) | 38 (40.4)^b^  52/145 (35.9)^b^  54 (57.4)^a^  45 (47.9)^b^  7/45 (15.6)  35/38 (92.1)  20 (57.1)  15 (42.9)  38 (38, 39)  20/35 (57.1)  15/35 (42.9)  3/35 (8.6)  2/35 (5.7)  1/35 (2.9)  3250 (2910, 3400)^a^  0.18 (-0.47, 0.50)  2/35 (5.7)  1/35 (2.9) | 52 (59.1)^a^  72/136 (52.9)^c^  71 (80.7)^b^  62 (70.5)^c^  8/62 (12.9)  45/52 (86.5)  21 (46.7)  24 (53.3)  38 (37, 39)  29/45 (64.4)  16/45 (35.6)  6/45 (13.3)  2/45 (4.4)  0  3140 (2770, 3385)^a^  -0.10 (-0.70, 0.42)  5/45 (11.1)  2/45 (4.4) | 37 (60.7)^a^  49/94 (52.1)^a,c^  43 (70.5)^a,b^  40 (65.6)^a,c^  2/40 (5.0)  33/37 (89.2)  18 (54.5)  15 (45.5)  38 (37, 39)  21/33 (63.6)  12/33 (36.4)  5/33 (15.2)  3/33 (9.1)  0  3100 (2840, 3265)^b^  -0.36 (-0.59, 0.14)  1/33 (3.0)  1/33 (3.0) | **0.021**  **0.007**  **0.002**  **0.005**  0.370  0.815  0.806  0.951  0.595  0.662  0.867  0.482  **0.038**  0.092  0.418  0.521 |

Data are presented as median (Q1, Q3) or number (percentage). FF, follicular fluid. *Among all singletons. ^a, b, c^ Different superscripts within the same line indicate statistical differences between subgroups. The quartiles of follicular oxytocin levels were 183.8 pg/mL (25th percentile), 257.7 pg/mL (50th percentile), and 345.0 (75th percentile).
