# Supplementary material for: Association between endogenous oxytocin levels and live birth rates following fresh embryo transfer: a prospective cohort study
Source: Front Endocrinol (Lausanne). 2026 Jun 2;17:1850346. doi: 10.3389/fendo.2026.1850346 (PMC13268967; doi:10.3389/fendo.2026.1850346)
Supplement: Supplementary file 3 [file DataSheet3.docx]

**Model 1 (Multivariable Logistic Regression):** A standard binary logistic regression model was constructed to adjust for baseline confounding. Covariates were selected based on baseline differences (*P* < 0.10) and clinical relevance (Table S1), incorporating age, BMI, AMH, causes of infertility, fasting insulin, duration of stimulation, endometrial thickness, serum estradiol and progesterone levels on hCG trigger day, and the number of embryos transferred.

The model formula is defined as:

$$g\left( E\left( Y \right) \right)=\beta_{0}+\beta_{a}A+\sum_{j=1}^{n} \gamma_{i}C_{1i}$$

where *g()* represents the logit link function, *Y* is the binary outcome (live birth), $\beta_{0}$ is the intercept, $\beta_{a}$ is the regression coefficient for the exposure *A* (oxytocin), and $\gamma_{i}$ represents the coefficients for the predefined confounding factors $C_{1i}$.

**Model 2 (Inverse Probability Weighting, IPW):** Because not all enrolled patients proceeded to fresh embryo transfer due to our predefined freeze-all strategy, potential selection bias was introduced. To mitigate this, Model 2 utilized IPW to create a pseudo-population balancing the baseline covariates between patients who did and did not undergo fresh transfer. Let *S* = 1 denote individuals who underwent fresh transfer and *S* = 0 denote those who did not. The selection weights (*IPW*1) conditional on transfer-predictive covariates (*C*_2_) were calculated as:

$$IPW1=\frac{1}{P(S|C_{2})}，S=0$$

$$IPW1=\frac{1}{1-P(S|C_{2})}，S=1$$

where *C*_2_ includes causes of infertility, AMH, BMI, stimulation duration, endometrial thickness, and serum estradiol and progesterone. Model 2 was then constructed by incorporating these weights into Model 1.

**Model 3 (Double Robust Model, DRM):** To maximize the validity of our causal inferences, a DRM was applied to simultaneously adjust for selection bias and exposure-related confounding. In addition to the selection weights (*IPW*1), inverse probability of exposure weights (*IPEW*) were computed to adjust for confounding related to oxytocin distribution ($IPEW=\frac{P(A)}{P(A|C_{1})}$). The final composite weight (*IPW*2) was derived by multiplying the selection and exposure weights ($IPW2=IPW1*IPEW$). In Model 3, *IPW*1 was replaced by *IPW*2, yielding unbiased effect estimates provided that either the selection model or the outcome model is correctly specified.
